# A Cost-Effectiveness Evaluation of Germline *BRCA1* and *BRCA2* Testing in UK Women with Ovarian Cancer

**DOI:** 10.1016/j.jval.2017.01.004

**Published:** 2017-04

**Authors:** Anthony Eccleston, Anthony Bentley, Matthew Dyer, Ann Strydom, Wim Vereecken, Angela George, Nazneen Rahman

**Affiliations:** 1 DRG Abacus, Bicester, Oxfordshire, UK; 2 AstraZeneca UK Ltd., Luton, Bedfordshire, UK; 3 Division of Genetics and Epidemiology, Institute of Cancer Research, London, UK; 4 Cancer Genetics Unit, The Royal Marsden NHS Foundation Trust, London, UK

**Keywords:** BRCA gene testing, breast cancer, cost-effectiveness, ovarian cancer

## Abstract

**Objectives:**

To evaluate the long-term cost-effectiveness of germline *BRCA1* and *BRCA2* (collectively termed “BRCA”) testing in women with epithelial ovarian cancer, and testing for the relevant mutation in first- and second-degree relatives of BRCA mutation–positive individuals, compared with no testing. Female BRCA mutation–positive relatives of patients with ovarian cancer could undergo risk-reducing mastectomy and/or bilateral salpingo-oophorectomy.

**Methods:**

A cost-effectiveness model was developed that included the risks of breast and ovarian cancer; the costs, utilities, and effects of risk-reducing surgery on cancer rates; and the costs, utilities, and mortality rates associated with cancer.

**Results:**

BRCA testing of all women with epithelial ovarian cancer each year is cost-effective at a UK willingness-to-pay threshold of £20,000/quality-adjusted life-year (QALY) compared with no testing, with an incremental cost-effectiveness ratio of £4,339/QALY. The result was primarily driven by fewer cases of breast cancer (142) and ovarian cancer (141) and associated reductions in mortality (77 fewer deaths) in relatives over the subsequent 50 years. Sensitivity analyses showed that the results were robust to variations in the input parameters. Probabilistic sensitivity analysis showed that the probability of germline BRCA mutation testing being cost-effective at a threshold of £20,000/QALY was 99.9%.

**Conclusions:**

Implementing germline BRCA testing in all patients with ovarian cancer would be cost-effective in the United Kingdom. The consequent reduction in future cases of breast and ovarian cancer in relatives of mutation–positive individuals would ease the burden of cancer treatments in subsequent years and result in significantly better outcomes and reduced mortality rates for these individuals.

## Introduction

Approximately 7000 new cases of ovarian cancer are diagnosed in the United Kingdom every year [1], [2], [3], [4], of which 13% to 16% are caused by a germline mutation in either the *BRCA1* or the *BRCA2* (collectively termed “BRCA”) gene [5], [6], [7], [8], [9]. Knowing a patient’s BRCA mutation status is becoming increasingly important for optimal ovarian cancer management, provision of information about response to chemotherapy, suitability for targeted agents such as polyadenosine diphosphate ribose polymerase (PARP) inhibitors, future cancer surveillance requirements, and overall prognosis [10], [11], [12], [13].

Women with a germline BRCA mutation have a 10% to 50% lifetime risk of ovarian cancer and a 40% to 85% lifetime risk of breast cancer [14], [15]. Because of this, relatives of BRCA mutation–positive individuals often undertake testing to find out whether they have inherited the family mutation. This knowledge is used to decide whether to have enhanced cancer surveillance and/or risk-reducing surgery (RRS). If they choose to have RRS, bilateral salpingo-oophorectomy (RRBSO) and/or mastectomy (RRM) can be undertaken. Equally importantly, relatives who have not inherited the BRCA mutation can be spared these interventions.

Access to BRCA testing for patients with ovarian cancer across the United Kingdom and Europe has been highly variable, with many centers using complex criteria to determine which patients should be offered testing. Historically, eligibility was primarily determined by family history of breast and/or ovarian cancer [16], [17], [18]. Nevertheless, patients with a germline BRCA mutation do not always have a relevant family history of breast or ovarian cancer [5], [6], [9], [19], and therefore using these criteria to determine testing eligibility is suboptimal.

The objective of this study was to determine the cost-effectiveness of providing germline BRCA mutation testing to all women with epithelial ovarian cancer in the United Kingdom, and the subsequent testing and management of their relatives who have a mutation. Of note, we have considered only germline BRCA mutations. The small proportion of ovarian cancer due to somatic BRCA mutations is not considered here; such mutations are not heritable and therefore do not have implications for relatives.

## Methods

### Model Overview

A patient-level simulation with annual cycles was developed in Microsoft Excel^®^. In the model, a simulated cohort of adult patients with ovarian cancer (index population) and their cancer-free family members transitioned to various health states at the beginning of each cycle, including no cancer (family members only, with different risks of developing cancer depending on whether they choose RRS), ovarian cancer, breast cancer (family members only), and both ovarian and breast cancer. The model outputs were costs and quality-adjusted life-years (QALYs), which were calculated for each individual and aggregated to provide an incremental cost-effectiveness ratio (ICER). The model also calculated the number of new cancer cases prevented and the number of lives saved. The flow of individuals through the model was based on defined characteristics, with the path determined by calculated time-to-events or annual risks when time-to-event could not be calculated. The model adopted a 50-year time horizon, a UK health service perspective was used, and discount rates of 3.5% were applied to costs and outcomes, in accordance with UK health technology assessment guidelines [20]. Costs and outcomes were applied when the corresponding event occurred, and because this model is a patient-level simulation, the model has a “memory” of each patient’s previous events. Costs and utilities were applied simultaneously using an additive and multiplicative approach, respectively.

The simulated index population consisted of 7,284 patients eligible for BRCA testing, which corresponds to the incidence of ovarian cancer in the United Kingdom in 2013 [4]. This population was included in two scenarios, BRCA testing or no BRCA testing, for the testing and nontesting arms.

Patients with a BRCA mutation entered the model (with mutation status known by testing or unknown in the nontesting arm). Patients who underwent BRCA testing but did not have a BRCA mutation did not enter the model, because there will be no difference in costs and outcomes between the testing and nontesting arms; the cost of testing these patients was, however, included. On the basis of published data, 13% of patients were assumed to have a BRCA mutation, 60% of which were assumed to have a *BRCA1* mutation and 40% a *BRCA2* mutation [5], [6], [7], [8], [9]. Sensitivity analyses were included to vary this rate between 10% and 16% (±25%). If patients in the testing arm had a BRCA mutation, their simulated first-degree relatives were tested. If the relative had a BRCA mutation, simulated second-degree relatives were also tested. The age of simulated relatives upon model entry was calculated in relation to the age of the index case, and those younger than 25 years were tested when they reached the age of 25 years.

**Fig. 1:**
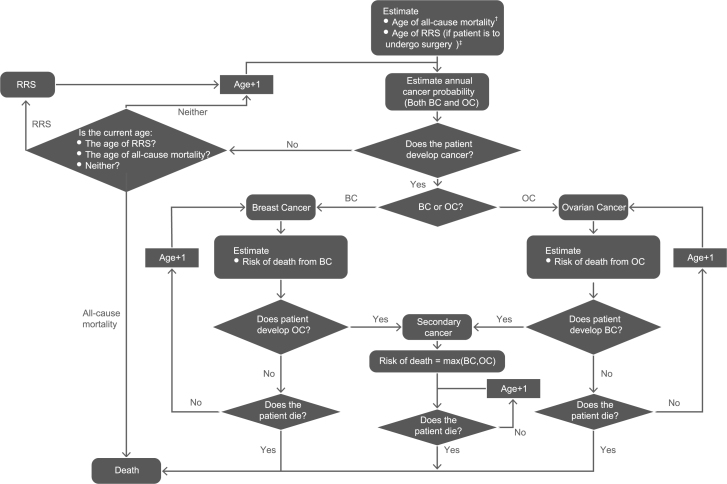
**Model schematic.** BC, breast cancer; OC, ovarian cancer; RRS, risk-reducing surgery. ^†^An age of all-cause mortality was estimated for each individual using UK national life tables, and an annual age-adjusted risk of death was estimated for individuals with cancer. ^‡^The age at which risk-reducing bilateral salpingo-oophorectomy occurred was assumed to be 40 years in *BRCA1* mutation–positive individuals and 45 years in *BRCA2* mutation–positive individuals, or on model entry for individuals older than these ages. The age of risk-reducing mastectomy was assumed to be 40 years or on model entry for older individuals.

The model schematic is shown in Figure 1. An age of all-cause mortality was estimated for each individual using UK national life tables [21], and an annual age-adjusted risk of death was estimated for individuals with cancer [22], [23]. Each year the model then determined whether individuals with cancer died from their cancer, until they reached their age of all-cause mortality.

RRS uptake was estimated using empirical data from the Royal Marsden Hospital of 858 women, 458 with a *BRCA1* and 400 with a *BRCA2* mutation. In *BRCA1* mutation carriers, the uptake of RRBSO was 88% and that of RRM was 34%. In *BRCA2* mutation carriers, the uptake of RRBSO was 87% and that of RRM was 25%. The uptake of RRBSO is slightly higher and the uptake of RRM slightly lower than published data [24], [25], and therefore different rates of RRS uptake were included in sensitivity analyses. The age at which RRBSO occurred was assumed to be 40 years in *BRCA1* mutation–positive individuals and 45 years in *BRCA2* mutation–positive individuals, or on model entry for individuals older than these ages. The age for RRM was assumed to be 40 years or on model entry for older individuals. The surgery cost and its impact on health-related quality of life (measured by a one-off disutility) were applied in the year that surgery took place. A hazard ratio (HR) was applied to the risk of cancer to reflect the lower risk after undergoing RRS.

When an individual developed cancer, treatment costs commenced and a risk of developing secondary cancer (breast/ovarian) was assigned. If secondary cancer developed, a new probability of age-adjusted cancer-related mortality was assigned.

Structural uncertainty within the model was addressed through standard modeling approaches and clinical validation to ensure that the patient pathway was captured accurately.

### Data Sources

Most of the data used in the model were UK-specific. Population data used to generate the model cohort are presented in Table 1. Published UK data were used to estimate the mean number of siblings and children [21]. All relatives generated were assigned an age according to the normal distribution and age relative to the index case (with an assumed SD of 5). Once an age was generated, life tables and random numbers were used to determine whether the individual was alive. The population was randomly generated using the probability of a first-degree relative being female as 50.78% [28]. The percentage of females in the generated cohort is slightly higher than this because the probability that the index patient’s mother is still alive is greater than for the father, because of a higher life expectancy in females than in males.

Cancer risk varied by age and BRCA mutation status (Table 2). A structured literature search was performed to identify the reduction in risk of breast cancer after RRM or RRBSO and the reduction in risk of ovarian cancer after RRBSO. There were eight relevant references [26], [27], [28], [29], [30], [31], [32], [33], the data from which were used in a fixed-effects meta-analysis to calculate the final HRs used in the model (Table 2). A fixed-effects method was used rather than a random-effects method because of low heterogeneity between studies. Only one publication [26] evaluated the risk reduction of breast cancer after both RRM and RRBSO. No evidence was identified to show that RRM affects the risk of ovarian cancer; therefore, for patients undergoing both RRM and RRBSO, the risk reduction of ovarian cancer after RRBSO was used.

The cancer-related mortality for both breast and ovarian cancer was estimated using 5-year net survival data reported by Cancer Research UK [22], [23], as presented in Table 2.

### Costs

Costs were included for BRCA testing, genetic counseling, cancer surveillance, RRS, hormone replacement therapy (HRT), cancer treatment, and palliative care (Table 3). HRT was included for individuals undergoing RRBSO without a history of breast cancer until the age of 52 years, as recommended by the National Institute for Health and Care Excellence (NICE) guidelines [17]. Cancer surveillance approaches (magnetic resonance imaging [MRI] or mammography per year) also followed NICE guidelines [17]. For genetic counseling, one post-test session for index patients with a BRCA mutation, one pretest genetic session for all relatives, and one additional post-test session for relatives found to have a BRCA mutation were included. This is in accordance with the mainstream model of genetic testing used at the Royal Marsden [9]. In sensitivity analyses, relatives received two pretest counseling sessions as recommended by NICE [17].

Costs for BRCA testing, genetic counseling, and RRS were applied in the cycle in which they occurred, whereas costs for HRT and surveillance (MRI and mammography) were applied annually; HRT costs were applied after RRBSO until the age of 52 years or the development of breast cancer, and MRI and mammography costs were applied after BRCA testing in BRCA mutation–positive patients until either breast or ovarian cancer developed.

Cancer treatment costs were derived from a microcosting exercise conducted in 2013 for the NICE familial breast cancer guideline [34]. Given the short life expectancy of those developing ovarian cancer and the high likelihood of repeat treatment, costs of treating ovarian cancer were applied annually. The survival rate for breast cancer is much greater, and therefore it was assumed that all treatment costs for breast cancer were applied for 1 year during the cycle when diagnosis occurred; nevertheless, it is acknowledged that breast cancer treatment may last longer. It was assumed that individuals who received a mastectomy before breast cancer diagnosis did not require surgery as part of their treatment; patients with ovarian cancer after RRBSO were, however, assumed to require additional debulking surgery in the year of diagnosis. Palliative care costs were applied in the cycle in which the patient died.

### Health State Utilities

Age-related utilities for females [35] were used in the model to ensure that the QALY gain associated with BRCA testing was not overestimated (Table 4).

The NICE clinical guideline 164 cost-effectiveness evidence review [36] provided utilities for both ovarian and breast cancer after diagnosis. These disease-specific utilities were combined with the age-related utilities multiplicatively as advised by the NICE Decision Support Unit [37], and the impact on quality of life was assumed to decrease each year after diagnosis until year 6, after which it remained constant. If a patient was diagnosed with both cancers, the utility values were also applied multiplicatively.

Utility values for the other health states and treatments in the model were derived from a time trade-off study in BRCA mutation–positive individuals [38]. This study reported that RRS was associated with a short-term detrimental impact on health-related quality of life and, consistent with UK clinical opinion, these utility values were assumed to apply only in the cycle in which RRS occurred. The base-case analysis applied no disutility for having a BRCA mutation, which is consistent with the Royal Marsden experience and other published studies [39], [40]. Nevertheless, a disutility has been reported in at least one study [38] and thus was included in sensitivity analyses. This disutility was assumed to apply for 1 year, on the basis of published evidence showing that the psychological impact of BRCA testing resolved within 1 year [41].

### Sensitivity Analyses

Parameter uncertainty around key model inputs was tested using sensitivity analyses, in which parameters were independently varied over a plausible range determined by either the 95% confidence interval (CI) or by clinical expert opinion; when no estimates were available, values were varied by ±25% of the corresponding base-case value (percentages were capped at 0% and 100%).

Joint parameter uncertainty was also explored through probabilistic sensitivity analysis (PSA), when all parameters were assigned distributions and varied jointly.

### Model Assumptions

There were a number of assumptions made during the development of the model:

• The sensitivity and specificity of full BRCA gene and specific mutation testing was 98%. This corresponds with the Royal Marsden empirical data and published literature [42], [43].
• Relatives with a BRCA mutation had the same BRCA mutation as the index case.
• Relatives considered in the model had no previous ovarian or breast cancer and had not undergone RRS.
• The 5- and 10-year risks for breast cancer and ovarian cancer, respectively, were constant over the 5 or 10 years. This is a simplifying assumption arising from the 5- and 10-year risk data used in the model for breast cancer and ovarian cancer.
• All RRMs were bilateral. This is a simplifying assumption arising because the HRs obtained from the literature were reported for patients receiving bilateral mastectomy.
• Patients did not develop both breast and ovarian cancer in the same year. This is a simplifying assumption supported by the Royal Marsden data. Although clinically possible, it is extremely unusual.
• The index population did not receive RRM. This is a simplifying assumption because RRM in patients with ovarian cancer with a BRCA mutation is rare.
• The costs and outcomes for patients without a BRCA mutation were equal between the testing and nontesting arms, because the risks of developing breast and/or ovarian cancer were the same for these patients in both arms. This means that the model considers only the incremental difference between testing and no testing in BRCA mutation–positive individuals (although the cost of testing individuals without a BRCA mutation was included).
• The population was not dynamic; therefore, the model did not consider relatives born after the index case was tested. This was a simplifying assumption because a dynamic population would have been impractically complex to model. Nevertheless, the approach taken allowed the results for testing an incident population from a single year to be assessed; the benefits of testing would be seen over the lifetime of these patients regardless of whether the testing scheme continued for longer than 1 year.
• The model was not a typical oncology cost-utility model and did not specifically consider cancer severity or treatments received (only one line of standard chemotherapy is considered in the model, and patients do not move to any other chemotherapy treatments [including targeted agents] and nor do they receive radiotherapy).

## Results

### UK Base Case

There were 7284 index cases run through the model, resulting in 3768 first-degree and 935 second-degree family members eligible for testing. In total, BRCA testing identified 1314 patients with a *BRCA1* mutation and 886 patients with a *BRCA2* mutation (Table 5).

The total discounted cost of BRCA testing in the arm that underwent testing (£9.6 million) was partially offset by a reduction in cancer treatment and palliative care costs, leading to an incremental discounted cost of £3.0 million. Over the 50-year time horizon, there were an additional 706 discounted QALYs associated with BRCA testing compared with no testing, resulting in an ICER of £4,339/QALY (95% CI £1,593–£11,764), which is lower than the UK threshold of £20,000/QALY. The cost-effectiveness plane for the base case is included in Appendix Figure 1 in Supplemental Materials found at doi:10.1016/j.jval.2017.01.004.

An important consequence of implementing BRCA testing in patients with ovarian cancer is the reduction in cancer and deaths among their relatives. If all women diagnosed with ovarian cancer were tested in 1 year, this analysis has calculated that there would be 77 fewer deaths, 141 fewer new cases of ovarian cancer, and 142 fewer new cases of breast cancer in relatives older than 50 years.

### Sensitivity Analyses

The results from the one-way sensitivity analyses did not differ substantially from the base case, and all results were lower than the UK cost-effectiveness threshold of £20,000/QALY. The cost-effectiveness plane for the PSA and a cost-effectiveness acceptability curve are included in Appendix Figures 2 and 3, respectively, in Supplemental Materials found at doi:10.1016/j.jval.2017.01.004, and the tabulated results of the individual one-way sensitivity analyses are included in Appendix Table 1 in Supplemental Materials found at doi:10.1016/j.jval.2017.01.004.

Changing the probability of having a BRCA mutation to 10% and 16% (base case 13%) had a small effect on the ICER (£5947 and £5800/QALY, respectively).

The RRBSO uptake rate in some published data [24], [25] is lower than the Royal Marsden data, and lowering the RRBSO uptake rate to 75% increased the ICER to £6139/QALY. Conversely, RRM uptake in published data is higher than the Royal Marsden data [24], [25]. Increasing the RRM uptake rate to 50% resulted in a slightly higher ICER (£5353/QALY) than the base case, because the higher costs of treatment were not offset by survival gains, because of high breast cancer survival in patients who do not undergo RRM.

Increasing the mean age of the index population to 60 years lowered the ICER to £3811/QALY. This was due to the generation of more grandchildren, and so there were more relatives receiving RRS and therefore more QALYs were accrued. Conversely, decreasing the mean age to 40 years increased the ICER to £4481/QALY.

Using the 95% CIs for the HR for the risk reduction in developing ovarian cancer after RRBSO resulted in ICERs that were similar to the base case (£3480 and £6449/QALY), whereas using the 95% CIs for the HR for developing breast cancer after RRM did not change the ICER (when accounting for rounding). This is because the CI ranges for RRM are very small and therefore have a very small effect on the ICER. Increasing the survival rates for breast cancer by 25% resulted in a higher ICER of £4442/QALY, whereas a decrease of 25% led to a lower ICER (£4165/QALY). Nevertheless, for ovarian cancer, 25% higher survival rates led to a lower ICER (£3458/QALY) and 25% lower survival resulted in a higher ICER (£5399/QALY).

Including two pretest genetic counseling sessions for relatives of the index population, as per NICE guidelines [17], slightly increased the ICER to £5094/QALY. When a disutility associated with BRCA testing of 0.87 was applied, this resulted in fewer QALYs gained (508) and a slightly higher ICER of £6026/QALY.

PSA (5,000 simulations of the cohort) showed that the expected ICER was £5,282/QALY (95% CI £1,593–£11,764). All simulation results were in the northeast or southeast quadrant of the cost-effectiveness plane, meaning that BRCA testing was always more effective than no testing. Overall, the probability of BRCA testing being cost-effective using a £20,000/QALY threshold was 99.9%. The inputs for the PSA are provided in Appendix Table 2 in Supplemental Materials found at doi:10.1016/j.jval.2017.01.004.

## Discussion

This study is an assessment of the cost-effectiveness of a novel pathway for integrating genetic testing into the routine clinical management of patients with ovarian cancer. In this model, testing can be undertaken in an existing oncology appointment, greatly reducing the testing turnaround time and cost associated with testing, allowing testing to be offered to a wider range of patients and relatives than is currently considered. The study shows that implementing routine BRCA testing in women with ovarian cancer would be cost-effective in the United Kingdom compared with no testing. It would result in lower breast and ovarian cancer incidence rates, lower treatment costs, lower cancer-related mortality, and an overall higher quality of life. The lives saved and the fewer new cases of ovarian and breast cancer in relatives in the testing arm are particularly important results in driving implementation.

NICE and the Cancer Strategy Taskforce recommend that patients with cancer at more than 10% risk of having a BRCA mutation should be offered testing [17], [44]. Several recent studies have shown that any woman with epithelial ovarian cancer is eligible by this criterion [5], [6], [7], [8], [9]. Many centers use family history of cancer to determine test eligibility, but this is much less effective in identifying women with BRCA mutations [45]. Some centers restrict testing to nonmucinous or high-grade serous ovarian cancer. Nevertheless, only approximately 3% of ovarian cancers are mucinous [46], some of which are due to other cancer predisposition genes that are frequently concurrently tested with *BRCA1* and *BRCA2*. Therefore, it is the simplest to offer testing to all women with epithelial ovarian cancer [47]. This would likely require some additional funding, although the increase in the number of tests will in part be offset by the substantial recent decrease in the cost of testing because of the use of new sequencing technologies [48]. Furthermore, as our results show, there will be longer term cost and health benefits. Although the aim of this analysis was to calculate the cost-effectiveness of BRCA testing versus no testing and therefore included all eligible patients, it is acknowledged that the uptake rate of BRCA testing may not be 100% in clinical practice.

It is interesting to note that a decrease in ovarian cancer survival rates leads to a higher ICER, and vice versa. This is because patients with ovarian cancer receive high-cost treatment for a shorter time because of lower survival rates, and therefore the cost savings associated with avoiding an ovarian cancer case are lower, despite a greater QALY gain for BRCA testing.

There were a number of limitations associated with the model and the data inputs used. First, this was not a typical oncology cost-utility model that tracks overall survival and progression-free survival, and it therefore did not specifically consider the treatments received apart from standard first-line chemotherapy, and no variation in cancer severity has been modeled because there were no data on the severity or stage of cancer at diagnosis. Nevertheless, the model used average survival rates for all cancer stages and average costs to reflect the impact of BRCA mutation testing and subsequent RRS. Over half of ovarian cancer cases are diagnosed at a late stage [49], meaning that the potential benefits seen with BRCA testing could be greater than the base-case results observed in this analysis.

Second, the simplified methodology of this model means that only the relatives of the index case benefit from BRCA testing and not the index population themselves because they entered the model with ovarian cancer and did not undergo RRM. (The results stratified by index population and their relatives are included in Appendix Tables 3 and 4 in Supplemental Materials found at doi:10.1016/j.jval.2017.01.004.) This is because ovarian cancer is almost always diagnosed at a late stage and survival rates are poor; therefore, patients are likely to receive chemotherapy treatment until their death, which prevents them from undergoing further surgery (e.g., RRM). Moreover, their mortality is nearly always determined by their ovarian cancer and not the risk of other (e.g., breast) cancer, with the remaining lifetime risk of another cancer being low because of poor survival at diagnosis of ovarian cancer. This approach is seen as a conservative assumption, because BRCA testing may benefit many patients with ovarian cancer in ways that are not captured in this model, such as providing information on the most appropriate chemotherapy choice for those with recurrent disease [5], [12]. In addition, BRCA mutation–positive patients with ovarian cancer are increasingly able to access targeted therapies such as PARP inhibitors, which have demonstrated benefit both as a single agent and as maintenance treatment in those with BRCA-mutated ovarian cancer [10], [50], [51]. PARP inhibitor therapies have also been shown to have activity in breast cancer [52], [53] and in male patients with BRCA mutation–positive prostate cancer [54], [55]. In our model, male first-degree relatives were tested for the BRCA mutation to identify any second-degree female relatives for testing; no benefit to them was, however, taken into account. The knowledge of BRCA mutation may provide patients with breast, ovarian, and prostate cancer access to targeted therapies that would not benefit patients without a BRCA mutation.

Third, no mortality or morbidity was considered for RRS; although this may bias the analysis in favor of testing, the rates of mortality and morbidity are generally low [28].

In the model, patients transition between health states at the beginning of each cycle, and the costs and utilities are assigned accordingly. The effect of transitioning between health states at different times in each cycle has not been investigated; nevertheless, it is not likely that this would have an impact on the results because of the long time horizon adopted in the model.

Patients with a BRCA mutation who choose not to receive RRS are still eligible for increased surveillance; NICE clinical guidance 164 for familial breast cancer recommends that BRCA mutation carriers aged 30 to 49 years should undergo annual MRI surveillance, and those older than 40 years should have annual mammograms [17]. It is important to note that it was not possible to capture the benefit or sensitivity of increased surveillance in terms of earlier diagnosis of cancer because the analysis did not specifically consider patients at different stages of their disease; the extra surveillance costs have, however, been included and therefore the results can be considered conservative.

The National Health Service also recommends screening mammograms every 3 years in all women aged 50 to 70 years [17]. This was not included in the nontesting arm of the model for women who had a BRCA mutation. Again, this can be seen as a conservative assumption, because including the costs of screening patients who were unaware of the mutation would increase the costs in the nontesting arm, and therefore reduce the incremental costs between the two arms and reduce the ICER, making BRCA testing even more cost-effective.

A previously published study by Kwon et al. [56] in 2010 estimated the cost-effectiveness of BRCA mutation testing in several different categories of patients with ovarian cancer in the United States and the downstream benefits for the first-degree relatives of patients with a BRCA mutation from the option of undergoing RRS. The study found that BRCA testing of women with ovarian cancer and a personal history of breast cancer, a family history of breast/ovarian cancer, or of Ashkenazi Jewish ancestry was cost-effective by preventing future breast and ovarian cancers among first-degree relatives with an ICER of $32,018 per life-year gained compared with no testing. This study cannot be directly compared with our results because of a number of differences; for example, the analysis was based on a US payer perspective, BRCA testing was performed only on patients with a personal or family history of cancer or Ashkenazi Jewish ancestry, and the pathway assessed was the traditional genetics referral model, which involves a patient with ovarian cancer being identified and referred to a clinical genetics service for pretest counseling, receiving test results, and post-test counseling (a much lengthier pathway with many more resources required, compared with this study). This study, however, also concluded that BRCA mutation testing with the option of RRS in relatives of patients with a BRCA mutation was cost-effective compared with no testing.

Another study by Manchanda et al. [57] estimated the cost-effectiveness of BRCA mutation testing in Ashkenazi Jewish women. Although this also is not directly comparable with this study, because it considered only a population with a much higher rate of mutation carriage than the general population, it also found that screening for BRCA mutations was highly cost-effective.

## Conclusions

The base-case analysis results show that germline BRCA mutation testing in women with epithelial ovarian cancer is cost-effective at a UK threshold of £20,000/QALY compared with no testing, with an ICER of £4,339/QALY (95% CI £1,593–£11,764). If all patients with ovarian cancer are tested in 1 year, there would be 141 fewer new cases of ovarian cancer, 142 fewer new cases of breast cancer, and 77 fewer deaths. These findings are robust to changes in the parameters, with all sensitivity analyses producing an ICER less than £20,000/QALY, and the probability that BRCA testing is cost-effective at this threshold is 99.9%. Implementing BRCA testing for all women with ovarian cancer would require some re-organization of testing services and may have some upfront resource implications; nevertheless, the reductions in the number of cases of both breast and ovarian cancer would ease the burden of cancer treatments in subsequent years and result in reduced mortality rates for these cancers.

## Figures and Tables

**Table 1:** Parameters for generating model cohort

**Index population**	**Data inputs**		**Number of patients**		**Reference**
Number of cases	7,284		7,284		ONS [1]
Age (y), mean ± SD*	50 ± 5		−		A normal distribution was applied to Domchek et al. [58]
BRCA mutation	13%		964		[5], [6], [7], [8], [9]
Proportion with *BRCA1* mutation	60%		583		
Proportion with *BRCA2* mutation	40%		381		
**First-degree relatives**†	**Mother**	**Father**	**Siblings**	**Children**	**Reference**
Number, mean ± SD*	1	1	0.91 ± 0.5	1.91 ± 0.5	ONS [59]
Age relative to index case, mean ± SD*	30 ± 5	32 ± 5	0 ± 5	−30 ± 5	A normal distribution was applied to ONS data [59]
Sex, probability female	100%	0%	50.78%	50.78%	ONS [60]
Probability BRCA mutation	50%	50%	50%	50%	Autosomal- dominant inheritance pattern
**Second-degree relatives**†	**Grandparents**	**Uncles/aunts**	**Nieces/nephews**	**Grandchildren**	**References**
Number, mean ± SD*	4	1.8 ± 0.5	1.7 ± 0.5	3.6 ± 0.5	Calculation based on ONS [59]
Age relative to first-degree relative, mean ± SD†	30 ± 5	0 ± 5	−30 ± 5	−30 ± 5	A normal distribution was applied to ONS data [59]
Sex, probability female	−	50.78%	50.78%	50.78%	ONS [60]
Probability BRCA mutation	25%	25%	25%	25%	Autosomal-dominant inheritance pattern

ONS, Office for National Statistics.

⁎ SDs were assumed.

† All individuals generated were assigned an age according to the normal distribution and age relative to the index case (with an SD of 5). Once an age was generated, life tables and random numbers were used to determine whether the individual was alive at the point of entry in the model.

**Table 2:** Cancer risks, risk reduction after RRS, and 5-y cancer survival rates

**Age range (y)**	***BRCA1***	***BRCA2***
*Approximate 5-y risk of breast cancer by age*		
20–25	5%	~1%
26–30	5%	2%
31–35	5%	5%
36–40	10%	2%
41–45	10%	10%
46–50	15%	10%
51–55	15%	10%
56–60	10%	10%
61–65	10%	15%
66–70	10%	15%
*Approximate 5-y risk of ovarian cancer by age*		
30–39	5%	5%
40–44	10%	5%
45–49	10%	10%
50–54	15%	10%
55–59	10%	10%
60–64	10%	5%
65–69	10%	5%
70–79	10%	5%
**RRS**	**Breast cancer HR (95% CI)**	**Ovarian cancer HR (95% CI)**
*HRs for the development of cancer after RRS (from meta-analysis)*		
*BRCA1*		
RRM	0.10 (0.03–0.31)	1.00
RRBSO	0.51 (0.39–0.66)	0.16 (0.09–0.26)
RRM and RRBSO	0.05 (0.01–0.22)	0.16 (0.09–0.26)
*BRCA2*		
RRM	0.09 (0.03–0.31)	1.00
RRBSO	0.39 (0.29–0.54)	0.12 (0.06–0.23)
RRM and RRBSO	0.05 (0.01–0.22)	0.12 (0.06–0.23)
**Age range (y)**	**5-y net survival of breast cancer (%)**	**5-y net survival of ovarian cancer (%)**
*5-y cancer survival rates*		
15–39	84.9	87.4
40–49	90.0	74.0
50–59	91.2	59.6
60–69	92.4	43.0
70–79	83.0	35.7
80–99	70.3	20.4

CI, confidence interval; HR, hazard ratio; RRBSO, risk-reducing bilateral salpingo-oophorectomy; RRM, risk-reducing mastectomy; RRS, risk-reducing surgery.

**Table 3:** Costs

**BRCA testing, RRS, and surveillance**	**Cost (£)**	**Reference**		
*BRCA testing*				
Index case (full genes)	306	Royal Marsden [61]		
Family members (specific mutation only)	108	Royal Marsden [61]		
Genetic counseling, per 2-h session	126	NICE CG164 [62]: On the basis of rate per hour of patient contact for band 7 counselor in primary medical care		
*RRS*				
Mastectomy including reconstructive surgery	9,219	NHS reference costs 2014–2015 [63]: Weighted average of HRG codes JA27Z and JA28Z		
BSO	2,976	NHS reference costs 2014–2015 MA08A–MA08B [63]		
HRT, per year	120.95	BNF 69 2015 [64] and HSCIC prescription cost analysis 2014 [65]: Weighted average of Kliovance, Evorel Conti, and Evorel Sequi		
*Surveillance*				
MRI, per year	191	NHS reference costs 2014–2015 [63]: HRG code RA05Z		
Mammography, per year	55	NICE CG144 costing report for venous thromboembolic diseases [66]; uplifted using PSSRU unit costs of health and social care 2014 [67]		
**Treatment**	**Unit cost (**£**)**	**Dose/units**	**Total cost (**£)	**Reference**
*Breast cancer*				
Breast surgery	3,186	1	3,816	NHS reference costs 2014–2015 [63]: Weighted average of HRG codes JA28A–C, JA39Z–JA41Z
Adjuvant radiotherapy	132	15	1,978	NHS reference costs 2014–2015 [63]: HRG code SC23Z
Chemotherapy delivery: first attendance	389	1	389	NHS reference costs 2014–2015 [63]: HRG code SB14Z
Chemotherapy delivery: subsequent attendance	326	5	1,632	NHS reference costs 2014–2015 [63]: HRG code SB15Z
Chemotherapy drugs (fluorouracil, epirubicin, cyclophosphamide)	205	6	1,230	BNF 69 2015 [64]
Neulasta*	686	6	4,118	BNF 69 2015 [64]
Dexamethasone†	0.78	16 mg OD for 2 days	12	BNF 69 2015 [64]
Anastrozole‡	0.07	1 mg OD for 5 y	Variable§	BNF 69 2015 [64]
Total with surgery			13,189	–
Total without surgery			9,373	–
*Ovarian cancer*				
Debulking surgery	5,613	1	5,613	NHS reference costs 2014–2015 MA26A–MA26C [63]
Chemotherapy delivery: first attendance	389	1	389	NHS reference costs 2014–2015 [63]: HRG code SB14Z
Chemotherapy delivery: subsequent attendance	326	5	1,632	NHS reference costs 2014–2015 [63]: HRG code SB15Z
Chemotherapy drugs (33% carboplatin, 67% carboplatin + paclitaxel)	568	6	3,408	BNF 69 2015 [64]
Neulasta*	668	6	4,118	BNF 69 2015 [64]
Dexamethasone†	0.78	16 mg OD for 2 days	12	BNF 69 2015 [64]
Total with surgery			15,185	–
Total without surgery			9,572	–
**Condition**	**Cost (**£**)**	**Reference**		
*Palliative care*				
Breast cancer	3,702	UK study of treatment patterns and resource costs for specific advanced cancer patients [68]; uplifted to 2013–2014 costs from PSSRU [67]		
Ovarian cancer	7,143			
All-cause mortality	103	NHS reference costs 2014–2015 [63]: HRG code SD03A		

BNF, British National Formulary; BSO, bilateral salpingo-oophorectomy; HRG, Healthcare Resource Group; HRT, hormone replacement therapy; HSCIC, Health and Social Care Information Centre; MRI, magnetic resonance imaging; NHS, National Health Service; NICE, National Institute for Health and Care Excellence; OD, one daily; PSSRU, Personal Social Services Research Unit; RRS, risk-reducing surgery.

⁎ Used to treat neutropenia to reduce the risk of infection.

† Used to treat inflammation, relieve sickness, and boost appetite.

‡ Used to inhibit the synthesis of estrogen as adjuvant treatment in estrogen-receptor– positive breast cancer.

§ The total cost of anastrozole varies between patients because some patients may die within the 5 years specified to receive this medication.

**Table 4:** Utility values

**Age (y)**	**Utility, mean ± SD**	
*Weighted health state index by age group in females*		
<25	0.94 ± 0.12	
25–34	0.93 ± 0.15	
35–44	0.91 ± 0.15	
45–54	0.85 ± 0.26	
55–64	0.81 ± 0.26	
65–74	0.78 ± 0.25	
≥75	0.71 ± 0.27	
**Time from diagnosis**	**Ovarian cancer**	**Breast cancer**
Cancer-related utilities*		
Year 1	0.50	0.71
Year 2	0.65	0.72
Year 3	0.67	0.73
Year 4	0.69	0.74
Year 5	0.70	0.76
Year 6+	0.72	0.77
**Health states**	**Utility values in controls, mean ± SD**	**Utility values in BRCA mutation carriers, mean ± SD**
*Health state utilities*		
Perfect health	1.00	1.00
RRM	0.88 ± 0.17	0.88 **±** 0.22
RRBSO	0.90 **±** 0.14	0.95 **±** 0.10
RRM and RRBSO	0.79 **±** 0.21	0.84 **±** 0.23
HRT	1.00	1.00
Healthy with a known BRCA mutation (sensitivity analysis only)	0.87 **±** 0.16	0.92 **±** 0.15
Death	0.00	0.00

HRT, hormone replacement therapy; NICE, National Institute for Health and Care Excellence; RRBSO, risk-reducing bilateral salpingo-oophorectomy; RRM, risk-reducing mastectomy.

⁎ Cancer-related utilities were derived from the NICE cost-effectiveness evidence review for familial breast cancer [36], where a steady improvement in quality of life was assumed to occur over the 5 years after diagnosis.

**Table 5:** Cost-effectiveness results

**Model outcome**	**Index population**	**First-degree relatives**	**Second-degree relatives**	**Total**
Number of patients	7,284	3,768	935	11,987
% Female	100%	54%*	100%	86%
Number with *BRCA1* mutation	583	592	139	1,314
Number with *BRCA2* mutation	381	411	94	886
	**No testing**	**BRCA testing**	**Difference**	
Costs (£)				
Testing	0	2,685,269	2,685,269	
Counseling	0	908,132	908,132	
RRM	0	2,487,991	2,487,991	
RRBSO	0	2,288,029	2,288,029	
HRT	0	298,329	298,329	
Surveillance costs	0	965,233	965,233	
Total testing costs	0	9,632,983	9,632,983	
Ovarian cancer treatment	85,720,007	80,588,951	–5,131,057	
Breast cancer treatment	4,536,269	3,500,468	–1,035,800	
Palliative care	6,577,195	6,172,490	–404,705	
Total discounted costs	96,833,471	99,894,892	3,061,420	
*Outcomes*				
Number dead	1,950	1,873	–77	
Number of ovarian cancer cases	1,218	1,077	–141	
Number of breast cancer cases	539	397	–142	
Total discounted QALYs	21,591	22,296	706	
ICER (95% CI)	£4,339/QALY (£1,593–£11,764)			

CI, confidence interval; HRT, hormone replacement therapy; ICER, incremental cost-effectiveness ratio; QALY, quality-adjusted life-year; RRBSO, risk-reducing bilateral salpingo-oophorectomy; RRM, risk-reducing mastectomy.

⁎ This population was randomly generated using the probability of a first-degree relative being female as 50.78%. The percentage female in the generated cohort is slightly higher than this because the probability that the index patient’s mother is still alive is greater than for the father, because of a higher life expectancy in females than in males.

## Footnotes

Supplemental material accompanying this article can be found in the online version as a hyperlink at doi:10.1016/j.jval.2017.01.004 or, if a hard copy of article, at www.valueinhealthjournal.com/issues (select volume, issue, and article).
