# Supplementary figures and images for: A Cost-Effectiveness Evaluation of Germline BRCA1 and BRCA2 Testing in UK Women with Ovarian Cancer
Source: Value Health. 2017 Apr;20(4):567–76. doi: 10.1016/j.jval.2017.01.004 (PMC5406158; doi:10.1016/j.jval.2017.01.004)

# Cost-effectiveness plane

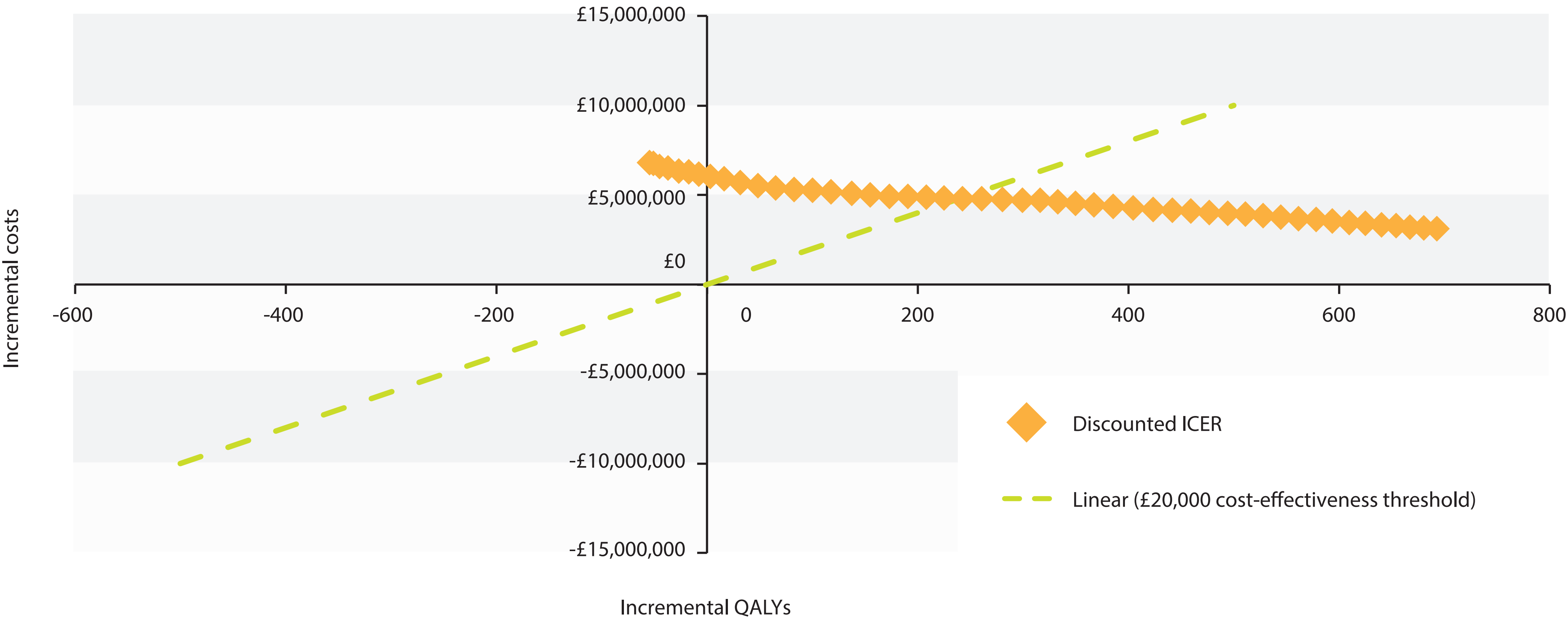

Supplement: Supplementary file 1 — Supplementary material [file mmc1.pdf]

# Cost-effectiveness plane

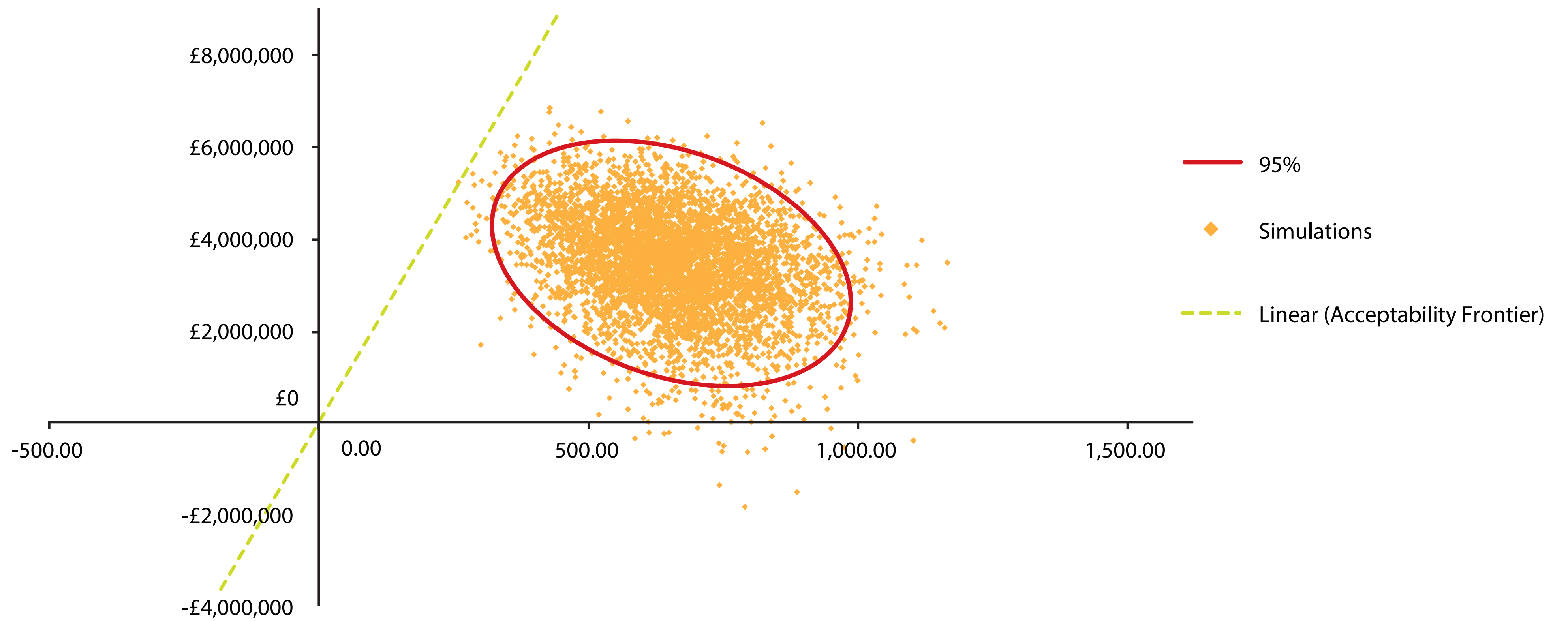

Supplement: Supplementary file 2 — Supplementary material [file mmc2.pdf]

# Cost-effectiveness acceptability curve

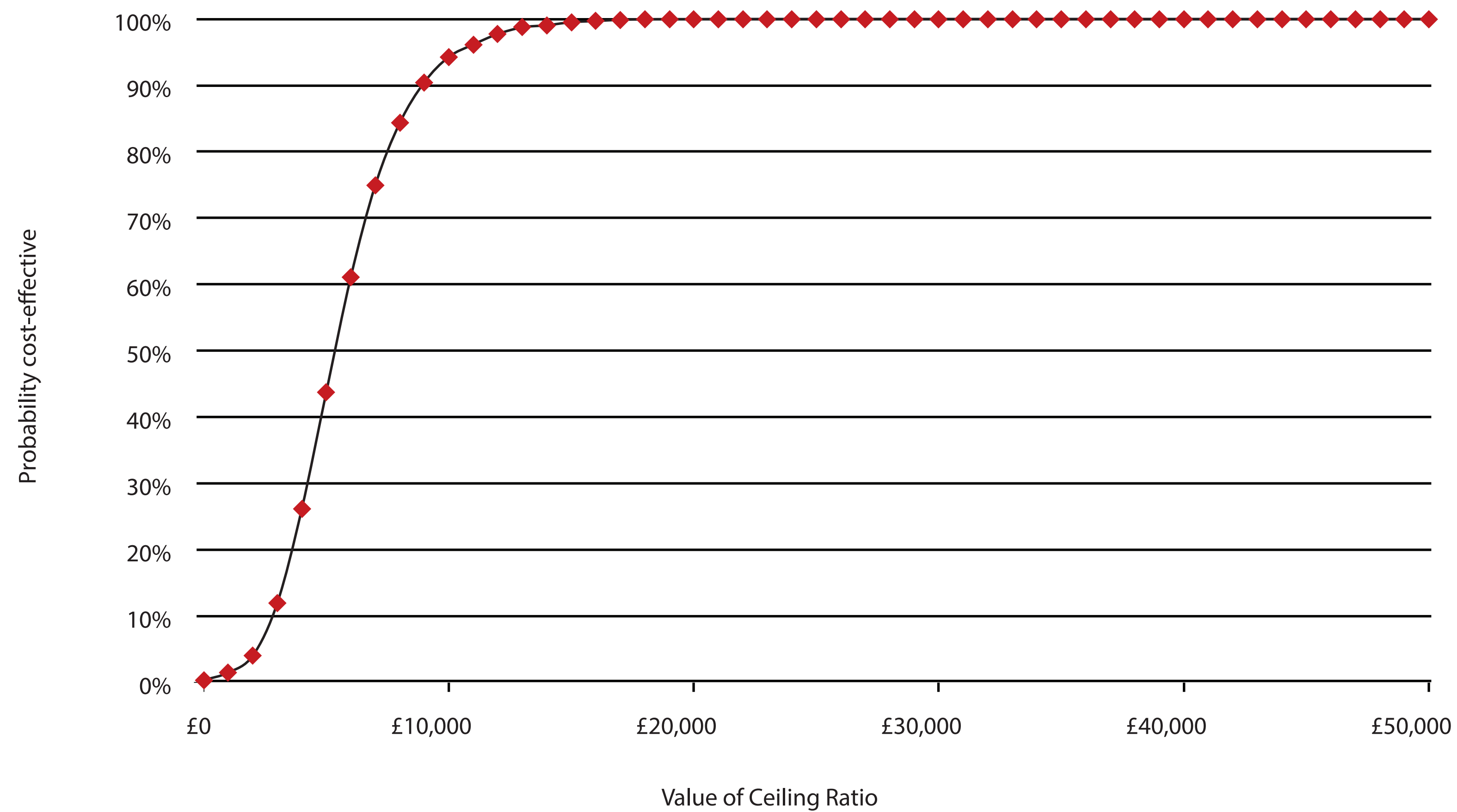

Supplement: Supplementary file 3 — Supplementary material [file mmc3.pdf]
