# Supplementary material for: A Cost-Effectiveness Evaluation of Germline BRCA1 and BRCA2 Testing in UK Women with Ovarian Cancer
Source: Value Health. 2017 Apr;20(4):567–76. doi: 10.1016/j.jval.2017.01.004 (PMC5406158; doi:10.1016/j.jval.2017.01.004)
Supplement: Supplementary file 4 — Supplementary material [file mmc4.docx]

# Supplementary information

Figure 1: Cost-effectiveness plane for the base case

Figure 2: Cost-effectiveness plane for the probabilistic sensitivity analysis

Figure 3: Cost-effectiveness acceptability curve
