# Supplementary material for: A Cost-Effectiveness Evaluation of Germline BRCA1 and BRCA2 Testing in UK Women with Ovarian Cancer
Source: Value Health. 2017 Apr;20(4):567–76. doi: 10.1016/j.jval.2017.01.004 (PMC5406158; doi:10.1016/j.jval.2017.01.004)
Supplement: Supplementary file 5 — Supplementary material [file mmc5.docx]

# Supplementary tables

Table 1: Results of the one-way scenario sensitivity analyses

| **Scenario** | **Incremental discounted costs** | **Incremental discounted QALYs** | **ICER (cost/QALY)** |
| --- | --- | --- | --- |
| Base case | £3,061,420 | 706 | £4,339 |
| Probability of BRCA mutation 10% | £3,110,430 | 523 | £5,947 |
| Probability of BRCA mutation 16% | £4,066,424 | 701 | £5,800 |
| RRBSO uptake 75% | £3,549,800 | 578 | £6,139 |
| RRM uptake 50% | £4,039,830 | 755 | £5,353 |
| Mean age of index case 60 | £2,816,096 | 739 | £3,811 |
| Mean age of index case 40 | £2,993,684 | 699 | £4,281 |
| RRBSO HR 95% CI LB | £2,717,755 | 781 | £3,480 |
| RRBSO HR 95% CI UB | £3,905,846 | 606 | £6,449 |
| RRM HR 95% CI LB | £3,061,420 | 706 | £4,339 |
| RRM HR 95% CI UB | £3,061,420 | 706 | £4,339 |
| RRM + RRBSO CI LB | £2,900,400 | 713 | £4,070 |
| 25% higher BC survival rates | £2,906,379 | 654 | £4,442 |
| 25% lower BC survival rates | £3,199,416 | 768 | £4,165 |
| 25% higher OC survival rates | £2,285,652 | 661 | £3,458 |
| 25% lower OC survival rates | £4,165,129 | 771 | £5,399 |
| Two pre-test genetic counselling sessions | £3,593,850 | 706 | £5,094 |
| BRCA positive result disutility of 0.87 | £3,061,420 | 508 | £6,026 |

Abbreviations: BC, breast cancer; CI, confidence interval; HR, hazard ratio; ICER, incremental cost-effectiveness ratio; LB, lower bound; OC, ovarian cancer; QALY, quality-adjusted life year; RRBSO, risk-reducing bilateral salpingo-oophorectomy; RRM, risk-reducing mastectomy; UB, upper bound.

Table 2: Inputs for probabilistic sensitivity analysis

| **Parameter** | **Base** | **SE** | **Distribution** |
| --- | --- | --- | --- |
| **Model setup** | | | |
| Discount rate (costs) | 3.5% | 0.004 | Beta |
| Discount rate (utilities) | 3.5% | 0.004 | Beta |
| Time horizon | 50 | – | None |
| **Ovarian cancer risk** | | | |
| 10 year risk BRCA1: Age 30–39 | 4% | 0.01 | Beta |
| 10 year risk BRCA1: Age 40–44 | 8% | 0.01 | Beta |
| 10 year risk BRCA1: Age 45–49 | 8% | 0.01 | Beta |
| 10 year risk BRCA1: Age 50–54 | 11% | 0.02 | Beta |
| 10 year risk BRCA1: Age 55–59 | 8% | 0.01 | Beta |
| 10 year risk BRCA1: Age 60–64 | 8% | 0.01 | Beta |
| 10 year risk BRCA1: Age 65–69 | 8% | 0.01 | Beta |
| 10 year risk BRCA1: Age 70–79 | 8% | 0.01 | Beta |
| 10 year risk BRCA2: Age 30–39 | 4% | 0.01 | Beta |
| 10 year risk BRCA2: Age 40–44 | 4% | 0.01 | Beta |
| 10 year risk BRCA2: Age 45–49 | 8% | 0.01 | Beta |
| 10 year risk BRCA2: Age 50–54 | 8% | 0.01 | Beta |
| 10 year risk BRCA2: Age 55–59 | 8% | 0.01 | Beta |
| 10 year risk BRCA2: Age 60–64 | 4% | 0.01 | Beta |
| 10 year risk BRCA2: Age 65–69 | 4% | 0.01 | Beta |
| 10 year risk BRCA2: Age 70–79 | 4% | 0.01 | Beta |
| **Breast cancer risk** | | | |
| 5 year risk BRCA1: Age 20–25 | 4% | 0.01 | Beta |
| 5 year risk BRCA1: Age 26–30 | 4% | 0.01 | Beta |
| 5 year risk BRCA1: Age 31–35 | 4% | 0.01 | Beta |
| 5 year risk BRCA1: Age 36–40 | 8% | 0.01 | Beta |
| 5 year risk BRCA1: Age 41–45 | 8% | 0.01 | Beta |
| 5 year risk BRCA1: Age 46–50 | 11% | 0.02 | Beta |
| 5 year risk BRCA1: Age 51–55 | 11% | 0.02 | Beta |
| 5 year risk BRCA1: Age 56–60 | 8% | 0.01 | Beta |
| 5 year risk BRCA1: Age 61–65 | 8% | 0.01 | Beta |
| 5 year risk BRCA1: Age 66–70 | 8% | 0.01 | Beta |
| 5 year risk BRCA2: Age 20–25 | 1% | 0.00 | Beta |
| 5 year risk BRCA2: Age 26–30 | 2% | 0.00 | Beta |
| 5 year risk BRCA2: Age 31–35 | 4% | 0.01 | Beta |
| 5 year risk BRCA2: Age 36–40 | 2% | 0.00 | Beta |
| 5 year risk BRCA2: Age 41–45 | 8% | 0.01 | Beta |
| 5 year risk BRCA2: Age 46–50 | 8% | 0.01 | Beta |
| 5 year risk BRCA2: Age 51–55 | 8% | 0.01 | Beta |
| 5 year risk BRCA2: Age 56–60 | 8% | 0.01 | Beta |
| 5 year risk BRCA2: Age 61–65 | 11% | 0.02 | Beta |
| 5 year risk BRCA2: Age 66–70 | 11% | 0.02 | Beta |
| **RRBSO** | | | |
| BRCA1: Estimated age of surgery | 40 | 5.10 | Normal |
| BRCA1: Uptake rate ≥ estimated age | 88% | 0.09 | Beta |
| BRCA2: Estimated age of surgery | 45 | 5.74 | Normal |
| BRCA2: Uptake rate ≥ estimated age | 87% | 0.09 | Beta |
| BRCA1: Ovarian cancer HR | 0.16 | 0.27 | Log-normal |
| BRCA1: Breast cancer HR | 0.51 | 0.13 | Log-normal |
| BRCA2: Ovarian cancer HR | 0.12 | 0.34 | Log-normal |
| BRCA2: Breast cancer HR | 0.39 | 0.16 | Log-normal |
| **RRM** | | | |
| BRCA1: Estimated age of surgery | 40 | 5.10 | Normal |
| BRCA1: Uptake rate ≥ estimated age | 34% | 0.04 | Beta |
| BRCA2: Estimated age of surgery | 40 | 5.10 | Normal |
| BRCA2: Uptake rate ≥ estimated age | 25% | 0.03 | Beta |
| BRCA1: Breast cancer HR | 0.10 | 0.60 | Log-normal |
| BRCA2: Breast cancer HR | 0.09 | 0.60 | Log-normal |
| **RRBSO + RRM** | | | |
| Ovarian cancer HR: BRCA1 | 0.16 | 0.27 | Log-normal |
| Breast cancer HR: BRCA1 | 0.05 | 0.79 | Log-normal |
| Ovarian cancer HR: BRCA2 | 0.12 | 0.34 | Log-normal |
| Breast cancer HR: BRCA2 | 0.05 | 0.79 | Log-normal |
| **Cumulative survival probability** | | | |
| Males: Age 0–101 | – | – | Beta |
| Females: Age 0–101 | – | – | Beta |
| **Ovarian cancer: Five-year survival rate** | | | |
| Age range 15–39 | 87.4% | 0.09 | Beta |
| Age range 40–49 | 74.0% | 0.09 | Beta |
| Age range 50–59 | 59.6% | 0.08 | Beta |
| Age range 60–69 | 43.0% | 0.05 | Beta |
| Age range 70–79 | 35.7% | 0.05 | Beta |
| Age range 80–99 | 20.4% | 0.03 | Beta |
| **Breast cancer: Five-year survival rate** | | | |
| Age range 15–39 | 84.9% | 0.09 | Beta |
| Age range 40–49 | 90.0% | 0.08 | Beta |
| Age range 50–59 | 91.2% | 0.08 | Beta |
| Age range 60–69 | 92.4% | 0.08 | Beta |
| Age range 70–79 | 83.0% | 0.10 | Beta |
| Age range 80–99 | 70.3% | 0.09 | Beta |
| **Costs** | | | |
| BRCA test: Index case | £306 | 39.03 | Gamma |
| Genetic counselling: Index case | £306 | 39.03 | Gamma |
| BRCA test: Family members | £126 | 16.07 | Gamma |
| Genetic counselling: Family members | £108 | 13.78 | Gamma |
| RRBSO total cost | £108 | 13.78 | Gamma |
| RRM total cost | £126 | 16.07 | Gamma |
| HRT annual cost | £2,976 | 379.56 | Gamma |
| Ovarian cancer: Cost with surgery | £9,219 | 1175.89 | Gamma |
| Breast cancer: Cost with surgery | £120.95 | 15.43 | Gamma |
| Ovarian cancer: Cost without surgery | £15,185 | 1936.87 | Gamma |
| Breast cancer: Cost without surgery | £13,189 | 1682.34 | Gamma |
| Palliative care: Ovarian cancer | £7,143 | 911.07 | Gamma |
| Palliative care: Breast cancer | £3,702 | 472.18 | Gamma |
| Palliative care: All-cause | £103 | 13.14 | Gamma |
| Anastrozole cost per day | £0.07 | 0.01 | Gamma |
| MRI scan total cost | £191 | 24.38 | Gamma |
| Mammography total cost | £55 | 6.95 | Gamma |
| **Utilities** | | | |
| Perfect health: Controls | 1.00 | – | None |
| RRBSO: Controls | 0.90 | 0.08 | Beta |
| RRM: Controls | 0.88 | 0.09 | Beta |
| RRM & RRBSO: Controls | 0.79 | 0.10 | Beta |
| HRT: Controls | 1.00 | – | None |
| BRCA+ test result: Controls | 1.00 | 0.06 | Beta |
| Death: Controls | 0.00 | – | None |
| Perfect health: Patients | 1.00 | – | None |
| RRBSO: Patients | 0.95 | 0.07 | Beta |
| RRM: Patients | 0.88 | 0.09 | Beta |
| RRM & RRBSO: Patients | 0.84 | 0.09 | Beta |
| HRT: Patients | 1.00 | – | None |
| BRCA+ test result: Patients | 1.00 | 0.06 | Beta |
| Death: Patients | 0.00 | – | None |
| Age-related utilities: Males age 1–101 | – | – | Beta |
| Age-related utilities: Females age 1–101 | – | – | Beta |
| Ovarian Cancer: Year 1 | 0.50 | 0.06 | Beta |
| Ovarian Cancer: Year 2 | 0.65 | 0.08 | Beta |
| Ovarian Cancer: Year 3 | 0.67 | 0.09 | Beta |
| Ovarian Cancer: Year 4 | 0.69 | 0.09 | Beta |
| Ovarian Cancer: Year 5 | 0.70 | 0.09 | Beta |
| Ovarian Cancer: Year 5+ | 0.72 | 0.09 | Beta |
| Breast Cancer: Year 1 | 0.71 | 0.09 | Beta |
| Breast Cancer: Year 2 | 0.72 | 0.09 | Beta |
| Breast Cancer: Year 3 | 0.73 | 0.09 | Beta |
| Breast Cancer: Year 4 | 0.74 | 0.09 | Beta |
| Breast Cancer: Year 5 | 0.76 | 0.10 | Beta |
| Breast Cancer: Year 5+ | 0.77 | 0.10 | Beta |
| **Duration of disutility** | | | |
| Duration of effect (yrs): Perfect health | – | – | None |
| Duration of effect (yrs): RRBSO | 1.00 | 0.06 | Log-normal |
| Duration of effect (yrs): RRM | 1.00 | 0.06 | Log-normal |
| Duration of effect (yrs): RRM & RRBSO | 1.00 | 0.06 | Log-normal |
| Duration of effect (yrs): HRT | ≤ age 52 | – | None |
| Duration of effect (yrs): BRCA mut positive test result | 1.00 | 0.06 | Log-normal |
| Duration of effect (yrs): Death | – | – | None |
| **Population generation** | | | |
| Number of index cases | 7,284 | – | None |
| Index population: Mean age | 50 | 6.38 | Normal |
| Index population: Age SD | 5 | – | None |
| Index population: Probability germline BRCA mut positive | 13% | 0.02 | Beta |
| Index population: % BRCA1 | 60% | 0.08 | Beta |
| First-degree: Probability of BRCA mutation if index case is BRCA mutation positive | 50% | 0.06 | Beta |
| Mother: Mean age (relative to index) | 30 | 3.83 | Normal |
| Mother: Age SD | 5 | – | None |
| Father: Mean age (relative to index) | 32 | 4.12 | Normal |
| Father: Age SD | 5 | – | None |
| Siblings: Mean Number | 0.91 | 0.13 | Log-normal |
| Siblings: Number SD | 0.5 | – | None |
| Siblings: Mean age (relative to index) | 0 | – | None |
| Siblings: Age SD | 5 | – | None |
| Siblings: Gender (probability female) | 50.78% | 0.06 | Beta |
| Children: Mean Number | 1.91 | – | None |
| Children: Number SD | 1 | 0.06 | None |
| Children: Mean age (relative to index) | –30 | 3.83 | Normal |
| Children: Age SD | 5 | – | None |
| Children: Gender (probability female) | 50.78% | 0.06 | Beta |
| Second-degree: Probability of BRCA mutation if index case is BRCA mutation positive | 25% | 0.03 | Beta |
| **Surveillance** | | | |
| MRI minimum age | 30 | 3.83 | Normal |
| MRI maximum age | 50 | 6.38 | Normal |
| Mammogram minimum age | 51 | – | – |
| Mammogram maximum age | 70 | 8.93 | Normal |

Abbreviations: HR, hazard ratio; HRT, hormone replacement therapy; MRI, magnetic resonance imaging; mut, mutation; RRBSO, risk-reducing bilateral salpingo-oophorectomy; RRM, risk-reducing mastectomy; SD, standard deviation; SE, standard error; yrs, years.

Table 3: Results for the index population

|  | **No testing** | **BRCA testing** | **Difference** |
| --- | --- | --- | --- |
| **Costs** | | | |
| Testing | £0 | £2,228,904 | £2,228,904 |
| Counselling | £0 | £133,434 | £133,434 |
| RRM | £0 | £0 | £0 |
| RRBSO | £0 | £0 | £0 |
| HRT | £0 | £0 | £0 |
| Surveillance costs | £0 | £0 | £0 |
| **Total testing costs** | **£0** | **£2,362,338** | **£2,362,338** |
| Ovarian cancer treatment | £73,811,672 | £73,811,672 | £0 |
| Breast cancer treatment | £1,906,362 | £1,906,362 | £0 |
| Palliative care | £5,730,960 | £5,730,960 | £0 |
| **Total discounted costs** | **£81,448,993** | **£83,811,331** | **£2,362,338** |
| **Outcomes** | | | |
| **Total discounted QALYs** | **4,688** | **4,688** | **0** |
| **ICER** | | | **–** |

Abbreviations: HRT, hormone replacement therapy; ICER, incremental cost-effectiveness ratio; QALY, quality-adjusted life year; RRBSO, risk-reducing bilateral salpingo-oophorectomy; RRM, risk-reducing mastectomy.

Table 4: Results for the relatives

|  | **No testing** | **BRCA testing** | **Difference** |
| --- | --- | --- | --- |
| **Costs** | | | |
| Testing | £0 | £456,365 | £456,365 |
| Counselling | £0 | £774,698 | £774,698 |
| RRM | £0 | £2,487,992 | £2,487,992 |
| RRBSO | £0 | £2,288,033 | £2,288,033 |
| HRT | £0 | £298,327 | £298,327 |
| Surveillance costs | £0 | £965,233 | £965,233 |
| **Total testing costs** | **£0** | **£7,270,648** | **£7,270,648** |
| Ovarian cancer treatment | £11,908,297 | £6,777,242 | –£5,131,055 |
| Breast cancer treatment | £2,629,906 | £1,594,106 | –£1,035,800 |
| Palliative care | £846,236 | £441,531 | –£404,705 |
| **Total discounted costs** | **£15,384,439** | **£16,083,526** | **£699,087** |
| **Outcomes** | | | |
| **Total discounted QALYs** | **4,688** | **4,688** | **706** |
| **ICER** | | | **£991/QALY** |
